# The MRI-Linear Accelerator Consortium: Evidence-Based Clinical Introduction of an Innovation in Radiation Oncology Connecting Researchers, Methodology, Data Collection, Quality Assurance, and Technical Development

**DOI:** 10.3389/fonc.2016.00215

**Published:** 2016-10-13

**Authors:** Linda G. W. Kerkmeijer, Clifton D. Fuller, Helena M. Verkooijen, Marcel Verheij, Ananya Choudhury, Kevin J. Harrington, Chris Schultz, Arjun Sahgal, Steven J. Frank, Joel Goldwein, Kevin J. Brown, Bruce D. Minsky, Marco van Vulpen, On Behalf of the MR-Linac Consortium Clinical Steering Committee

**Affiliations:** ^1^Radiation Oncology, University Medical Center Utrecht, Utrecht, Netherlands; ^2^Radiation Oncology, MD Anderson Cancer Center Houston, Houston, TX, USA; ^3^Imaging Division, University Medical Center Utrecht, Utrecht, Netherlands; ^4^Radiation Oncology, Netherlands Cancer Institute, Antoni van Leeuwenhoek Hospital, Amsterdam, Netherlands; ^5^Clinical Oncology, Manchester Cancer Research Centre, The Christie Hospital, Manchester, UK; ^6^Clinical Oncology, Manchester Academic Health Sciences Centre, The Christie Hospital, Manchester, UK; ^7^Radiation Oncology, NIHR Biomedical Research Centre, The Institute of Cancer Research, The Royal Marsden Hospital, London, UK; ^8^Radiation Oncology, Froedtert and Medical College of Wisconsin, Milwaukee, WI, USA; ^9^Radiation Oncology, Sunnybrook Hospital, Toronto, ON, Canada; ^10^Elekta A.B., Stockholm, Sweden

**Keywords:** MR-linac, MRI, linear accelerator, radiotherapy, consortium, innovation

## Abstract

An international research consortium has been formed to facilitate evidence-based introduction of MR-guided radiotherapy (MR-linac) and to address how the MR-linac could be used to achieve an optimized radiation treatment approach to improve patients’ survival, local, and regional tumor control and quality of life. The present paper describes the organizational structure of the clinical part of the MR-linac consortium. Furthermore, it elucidates why collaboration on this large project is necessary, and how a central data registry program will be implemented.

## Clinical Introduction of MR-linac and Rationale

The development of image-guided radiotherapy (IGRT) has driven a paradigm shift in radiation oncology. Before the use of IGRT, large margins to correct for geometric uncertainty in space and time were required, with significant associated toxicity. Nowadays, a more precise and, for some tumor sites, ablative-type treatment approach has become standard of care with the additional goal of limiting toxicity and improving the therapeutic index. One of the major developments has been the introduction of on-board online cone-beam computed tomography (CBCT) imaging. For example, for tumors located in the lungs, this has had a dramatic impact on radiotherapy treatments, as the air–tissue interface provides optimal visibility of tumors and allows for a very precise and high-dose radiation delivery. This image-guided stereotactic body radiotherapy (SBRT) approach has resulted in major improvements in radiotherapy control rates and is now considered as a treatment option comparable to surgery (1). However, CBCT can only be used preceding, not during, treatment and does not allow for optimal imaging of tumors and organs at-risk when surrounded by soft tissues.

The MR-linac is a hybrid linear accelerator (linac) combined with a magnetic resonance imaging (MRI) scanner. As MRI can provide optimal soft-tissue contrast, the 1.5-T MR-linac can be used for image guidance in multiple sites throughout the body providing diagnostic quality images during treatment delivery and, therefore, allowing for very accurate image-guided daily adaptive radiotherapy. The technology has been described previously (2, 3). Among the expected additional benefits of the MR-linac compared with existing technologies is hypofractionated dose escalation to the tumor, aiming to increase local control rates and survival while having equal or decreased toxicity rates. Another approach would be to maintain the conventional dose and decrease the risk of toxicity by reducing margins for uncertainty and tumor motion, as the system allows for daily fast adaptive replanning and gated or tracked radiotherapy treatments. Furthermore, functional imaging may allow for adaptive focal boosting and personalized inhomogeneous target dosage based on response.

The first technical prototype MR-linac has been developed and installed in UMC Utrecht. Since 2008, it has been used to develop, evaluate, and validate clinical procedures, in collaboration with Elekta and Philips. In 2012, an international consortium (MR-linac Consortium) was formed, which will collaboratively implement the clinical introduction of the MR-linac. The MR-linac Consortium members include the University Medical Center Utrecht, The Netherlands; Netherlands Cancer Institute – Antoni van Leeuwenhoek Hospital, The Netherlands; The Institute of Cancer Research/The Royal Marsden Hospital, UK; The Christie NHS Foundation Trust/Manchester Cancer Research Centre, UK; The University of Texas MD Anderson Cancer Center, USA; The Froedtert & Medical College Wisconsin, USA; Sunnybrook Odette Cancer Center, Canada; Elekta AB, Sweden and Philips, The Netherlands. All consortium institutes will install clinical prototypes of the MR-linac. The first clinical prototypes have been installed at the UMC Utrecht and MD Anderson and are currently being commissioned and used to validate clinical procedures. Installation is planned for the other collaborating institutes in 2016. The research consortium has access to the developing MR-linac technology, for which extended technical specifications will be available at each clinical update (including the possibility of functional imaging).

**Figure 1:**
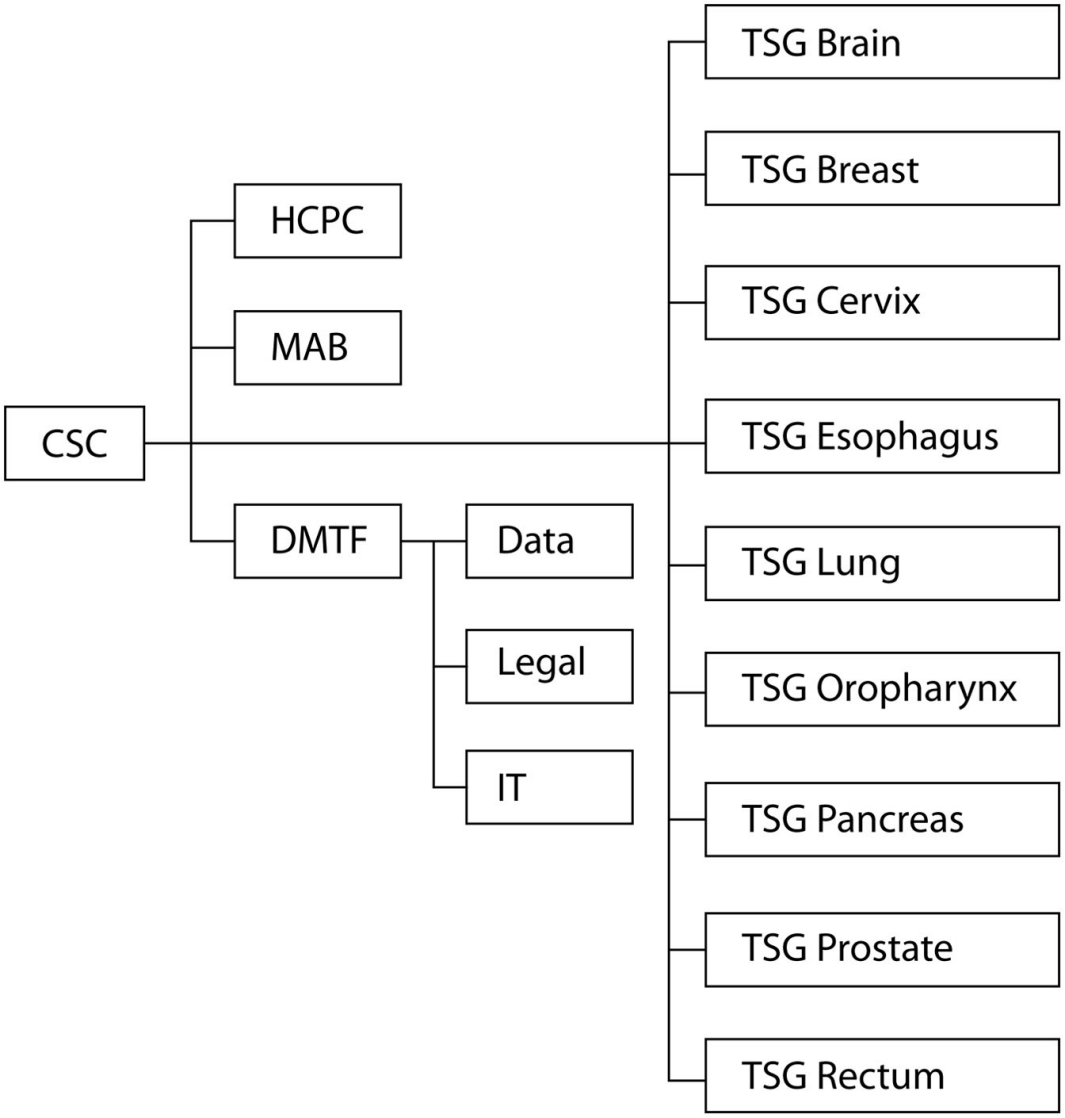
**Organizational structure of the clinical working groups in the MR-linac Consortium**. CSC, Clinical Steering Committee; HCPC, Health Care Policy Committee; MAB, Methodology Advisory Board; DMTF, Data Management Task Force; TSG, Tumor Site Group.

The goal of the present paper is to describe the organization of the MR-linac research consortium (Figure 1). The described structure may be adapted for implementation and evaluation of future technologies.

## Collaboration in MR-linac Consortium

**Figure 2:**
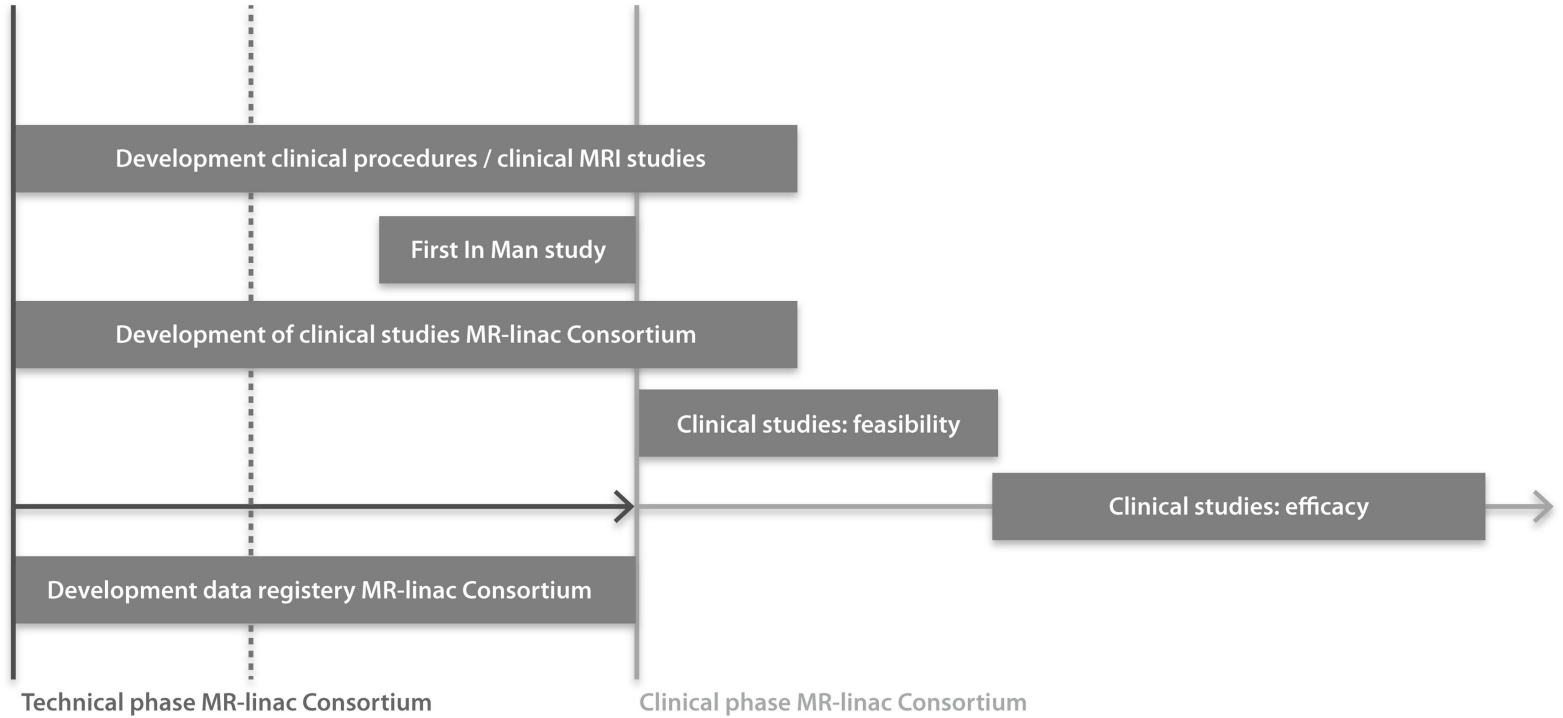
**Timeline of clinical studies within the MR-linac Consortium**. Installation of MR-linacs at all consortium institutes are either ongoing or planned for 2016. Regulatory approval for the integrated MR-linac system is planned for 2017.

Collaboration is essential for success in improving outcomes of radiation treatments when introducing an innovative and complex technology. The key role of the research consortium is to design studies and the data registry system in such a way that evidence will be gathered for maximizing and proving the clinical benefit of the MR-linac. This ambitious project aims to create a paradigm shift in radiation oncology to improve clinical outcomes. Therefore, the project is heavily dependent on the collaboration of the institutes. To achieve this, the consortium’s success is put first, rather than the interests of individuals. Having a consortium allows for mentorship of junior faculty by experienced senior faculty and will allow junior faculty to expand their knowledge and competencies. The multi-institutional studies performed within the consortium will allow for faster accrual of patients and larger patient numbers, thus providing evidence of the clinical benefit of the technology in a coordinated and more timely manner. In addition, the approval by regulatory bodies (CE, FDA, ethical committee) will be more straightforward and faster, and grant applications for national and international funding for clinical studies will be facilitated. Clinical working groups (WGs) will address and develop tumor site-specific MR-procedures and sequences, feasibility and efficacy studies (Figure 2). Pretreatment anonymized data and quality assurance (QA) data will be developed together and shared, shortening the time toward clinical implementation. By developing consortium-wide imaging protocols, consensus MR-based contouring atlases, dose prescription definitions and dose constraints, toxicity, quality of life (QoL) and patient-reported outcome measures (PROMS) scoring systems, and treatment registration parameters, the MR-linac will achieve consistency in radiation treatment and establish standards on quality. With all consortium members using standard, predefined survival parameters, criteria for tumor response and disease recurrence, toxicity criteria, and PROMS, multi-institutional clinical research into the effectiveness of the MR-linac will be easier and of higher quality. Trial approval processes may be time consuming, with associated costs and delay adoption. Submitting study proposals in a more structured way for multiple tumor sites at one time, i.e., an “umbrella” proposal, for Ethical Committee review, will facilitate an efficient and more straightforward approval process. Disadvantages of having a large research consortium including >100 radiation oncologists and >200 physicists, technologists, engineers, dosimetrists, radiation therapists, researchers, epidemiologists, and statisticians, are that communication requires an effective coordination including infrastructure, roles and responsibilities of the individuals involved. The number of consortium partners may increase the risk of slowing the trajectory from idea to clinical implementation and may impact the decision-making process. Furthermore, there are obviously costs associated with such a large organization, in terms of personnel, infrastructure, and meetings.

## Organizational Structure MR-linac Consortium

The consortium consists of technical and clinical WGs, which interact on a regular basis during group-specific meetings, consortium meetings, consortium-wide teleconferences, institute-specific meetings, and interval meetings *via* the coordinators of the WGs. The present manuscript will focus on the organization of the clinical WGs of the MR-linac consortium. The technical WGs are outside the scope of this paper, although extensively collaborating. An alternative organizational structure would have been to have a natural formation of clinical groups, although this would have resulted in a slower formation of groups, a larger number of tumor sites, risking delays in demonstrating clinical benefit.

### Clinical Steering Committee

The goal of the Clinical Steering Committee (CSC) is to stimulate and guide collaboration. The CSC discusses high-level aspects of clinical research, including study design, data management, organization, and collaboration. Individual members are responsible for communication within their respective institutions. Furthermore, the CSC has organized the development of the clinical WGs and has been involved in the selection of the first tumor sites for consortium-wide studies. After site-visits and meetings with department heads, clinicians, physicists, and industry, the CSC has been convened. The CSC has formed the clinical WGs, including anticipated participants, coordinators, roles, responsibilities, organizational structure, and communication pathways of the consortium. The CSC serves a key role in data oversight. The CSC will work closely with the other clinical groups to ensure that there is adherence to the collaboration agreements as well as to evaluate whether existing guidelines and agreements are relevant. Lastly, the CSC will manage data ownership, publication rights, and data release. As the decisions made by the CSC relate to department leadership decisions, the members are heads of their Radiation Oncology departments or act on behalf of the department heads. Any formal collaboration agreements are signed by the institute’s leadership.

### Tumor Site Groups

There are many clinical indications where the MR-linac may have an expected benefit (a consortium-wide questionnaire resulted in 25 candidate indications). When introducing a new technology in radiation oncology, it is important that the number of indications is not too high in the initial phases, since this can reduce the quality of research and delay clinical adoption. Therefore, nine tumor sites have been selected in which to start the consortium-wide clinical studies. This selection was based on the expected clinical benefits by the key opinion leaders (either increased local control or survival, or decreased toxicity, or improved QoL), potential patient numbers (average incidence per year) at the collaborating institutes, and the (tumor site specific) research expertise of the collaborating centers. The first tumor sites are a starting point and are intended not to exclude subsequent studies for other clinical indications. These nine consortium-wide tumor sites are brain, breast, cervix, esophagus, lung, oropharynx, pancreas, prostate, and rectum. Each institute coordinates one or more Tumor Site Groups (TSGs). The TSGs are chaired by a Disease Site Coordinator (DSC). The DSC facilitates trials on a tumor site, organizes, and supports the TSG, helps identify and secure adequate funding, and communicates with other clinical and technical WGs in the consortium. In each TSG, one physician participates from each institute, as well as physicists. The roles of the TSG are to discuss and prepare predicate clinical study proposals for MRI studies (i.e., MR-sequence optimization for radiotherapy, MR-based planning study, MR-based contouring study, and development of consensus delineation atlases), to prepare joint clinical study proposals for feasibility and future randomized controlled trials. The TSG also communicates with other WGs on the necessary technical specifications and anticipated timelines. As recommended by the Accelerated Access Review (aiming to accelerate access to innovations for NHS patients in the UK), patients should be involved in every step of the innovation pathway (4). Patients will be involved in the clinical trial development and evaluation for each disease site, leading to implementation of innovative treatments focusing on treatment outcomes that are most important to patients.

### Health Care Policy Committee

The HCPC will develop a comprehensive strategic plan to ensure patient access and reimbursement by communicating the value of treatment with the MR-linac by disease site to the appropriate agencies and stakeholders. The HCPC focuses on the MR-linac overall cost effectiveness for patients, health care providers, and funding bodies. The HCPC will work closely with the CSC and TSG to ensure that publications of the disease-site specific data are communicated in a timely manner. Gathering clinical evidence to establish the value of the treatment with the MR-linac (which will result from the efforts of the TSGs) will be a necessary step toward reimbursement. The WG activities will involve members of the consortium, patients, industry, medical societies, government, and managed care. The HCPC will evaluate the health care policies toward advanced technologies and the MR-linac by local governments, managed care, and medical societies.

### Data Management Task Force

All patients treated on the MR-linac by the Consortium members will be registered in a consortium-wide data registry and all clinical trials will be registered prospectively. Currently, most patients treated by radiotherapy are not enrolled in clinical trials. Furthermore, inefficient data management is a concern, as this may lead to loss of a significant amount of research data. The data registry ensures accurate and comprehensive data collection for evaluating and modeling treatment outcomes, radiomics, QA, and technological improvements. In addition, the joint data registry connects the institutes, strengthens their commitment to collaboration and will enhance transparency in the conduct and reporting of clinical trials. The clinical institutes of the consortium will have access to de-identified data and all anonymized clinical data. The data management WG “core team” is the DMTF, which has representatives from all institutes. In addition, three sub-teams have been installed; legal, IT, and medical/data in accordance with the guidelines by Skripcak et al. (5). The DMTF has prepared a model for data management, which has been approved by the CSC. The DMTF will propose the strategy, together with the required governance agreement to the institutes’ leadership, for final approval. After approval, the data registry will be implemented and tested at all institutes, before official release together with the associated documentation. The legal team will address aspects, such as privacy, governance documentation, data ownership, and will advise on the “umbrella” Ethical Committee protocol submission, as required prior to the start of inclusion of patients in the consortium-wide data registry. The IT team is developing the data registry system and methods to retrieve data from the various electronic hospital information systems and protected data storage. The data-team focuses on the classes of data to be collected, such as clinical information and tumor characteristics, imaging data, treatment plan data, machine treatment data, treatment outcomes (tumor control, survival, toxicity, PROMS). Each institute will appoint dedicated data managers to facilitate consortium-wide data collection.

### Methodology Advisory Board and the R-IDEAL Framework

The Methodology Advisory Board (MAB) advises on study design of MR-linac technology within clinical studies, aiming for evidence-based introduction of the technology. Past experiences have shown that the window of opportunity for appropriate evaluation of innovations in radiation oncology is narrow: differences in perception of equipoise and pressure from patients, doctors, and industry have led to implementation of innovations without robust clinical evidence of superiority. The MR-linac technology is complex, allows for numerous treatment strategies, and continuous technical improvements. As evaluation of complex interventions is challenging, the MAB has proposed to implement an adapted version of the IDEAL recommendations for evaluation of MR-linac (6), the IDEAL framework was originally developed for evaluation of surgical innovations and aims to prevent delay in the introduction of promising interventions without exposing patients to unnecessary risks and toxicity. IDEAL consists of five stages: an Idea or Innovation, which then undergoes Development and Exploration, and subsequently Assessment and Long-term studies. At each stage, the IDEAL framework includes recommendations for methodology and reporting. For radiotherapy innovations, we have added a stage 0 (radiotherapy predicate studies), covering all the work that is performed before the innovation is used for treating patients (such as delineation studies, optimization of MRI sequencing protocols – for instance, MRI sequence optimization for radiotherapy, MR-based planning study, planning study in the presence of 1.5T magnetic field, MR-only planning – and modeling studies to identify disease sites/indications where the MR-linac is expected to be of greatest benefit). For each tumor site and indication, for which, the MR-linac will be used, all stages need to be considered. After each stage, an assessment will take place in which it is decided whether the technique is ready to be taken to the next stage or whether previous steps need to be repeated. By recommending all TSGs to follow the R-IDEAL framework and to report results after each stage, we guarantee a high-quality, systematic, and uniform approach to evaluation and provide the opportunity for TSGs to learn from each other. The MAB interacts closely with the TSGs to advise on optimal study design at the different stages and gives feedback from study experiences from other groups.

## Interdisciplinary Collaboration and Technical Working Groups in the MR-linac Consortium

The quality of patient care in radiotherapy relies heavily on the teamwork of all members of the radiation oncology department. The team includes radiation oncologists, radiation physicists, dosimetrists, radiation therapists, nurses, support staff members, and clerical staff members. For the MR-linac project, there are additional interdisciplinary and international collaborations that will be essential if we are to accomplish safe and effective radiation treatment on this new platform. These individuals include technicians/radiographers, researchers, industry, IT, data managers, and members of the clinical and technical MR-linac Consortium WGs.

The uniqueness of the consortium lies in the early involvement of institutes in the development process of this innovative technology. The focus of industry is not only on solving engineering problems but also a long-term strategy for the route to adoption of the technology. This can only be achieved through generation of clinical evidence. When clinical members are not involved early, this results in adoption problems by the clinical community later, commonly at the phase where the community requires evidence a few years after the technology has been introduced. The advantage of the early collaboration of academia with industry is that working together on technology development and optimization ensures a more rapid availability of technical specifications to the institutes. In addition, the clinical members’ advise the developers on which specifications are required to optimally improve clinical outcomes for patients. This early collaboration does come with the potential risk of introduction of bias in the clinical results. To mitigate this risk and in order to maintain scientific integrity, the scientific evaluation of the clinical benefit of the technology will be done by the institutes themselves and will be independent from industry.

The four TWGs (i.e., QA, MRI, Adaptive Elements, and Workflow) are beyond the scope of the present clinical perspective paper. However, these groups collaborate closely with the clinical groups and interact on a regular basis and will therefore be described briefly below. In the TWGs, members from the industry and all institutes are collaborating. The QA group is responsible to implement a QA protocol following a Failure Mode Effect Analysis (FMEA) risk based approach. Subjects, such as reference dosimetry, offline and real-time QA, End2End QA, Patient QA, and MRI-QA, are reviewed and modified to ensure an optimal dosimetric and geometric accuracy of the entire system. The MRI group works on the optimization of MRI protocols, development of MR sequences, and MRI QA, including geometric accuracy. In the Adaptive Elements group, fast adaptive replanning, gating, and tracking solutions are developed. The Workflow group handles the technology-user interface, ensuring that the system is designed to support an efficient and effective clinical workflow for treatment of patients.

In summary, we report the development of a consortium based research group to help facilitate the design, implementation, and management of clinical research in MR-linac technology. The consortium provides a shared governance model, which is unique among academic centers. It engages broad membership and provides a clinical development plan to address outcomes and value of a new technology. The early involvement of institutes in the development process of the technology should increase the chance for success of this innovative technology.

## Author Contributions

LK, CF, HV, MV, AC, KH, CS, AS, SF, JG, KB, BM, and MV all had a substantial contribution to the conception or design of the work; drafting the work or revising it critically for important intellectual content; final approval of the version to be published.

## Conflict of Interest Statement

Elekta and Philips are members of the MR-linac Consortium. Elekta A.B. financially supports the MR-Linac Consortium and all the member institutes. Dr. CF, Dr. HV, Dr. MV, Dr. AC, Dr. KH, Dr. CS, Dr. BM, and Dr. MV declare no personal conflicts of interest. Dr. LK reports a grant from Elekta A.B. to UMC Utrecht. Dr. AS reports past educational seminars with Medtronic, Elekta and Varian medical systems, consulting/advisory role with Varian medical systems, research grant with Elekta A.B., Travel accommodations/expenses by Medtronic, Elekta and Varian. Dr. SF reports grants and other from Varian, grants and other from NIH/NCI, grants from P01, grants from U19, other from The University of Texas MD Anderson Cancer Center, other from IBA, other from Hitachi, other from ABR, other from ABS, other from C4 Imaging, outside the submitted work. In addition, Dr. SF has a patent MRI Markers issued. Dr. JG and Dr. KB report that they are employed by Elekta.
